# Effect of birthweight measurement quality improvement on low birthweight prevalence in rural Ethiopia

**DOI:** 10.1186/s12963-021-00265-0

**Published:** 2021-09-22

**Authors:** Estifanos Baye, Firehiwot Workneh Abate, Michelle Eglovitch, Fisseha Shiferie, Ingrid E. Olson, Tigest Shifraw, Workagegnehu Tarekegn Kidane, Kalkidan Yibeltal, Sitota Tsegaye, Mulatu Melese Derebe, Sheila Isanaka, Blair J. Wylie, Rose L. Molina, Grace J. Chan, Amare Worku, Luke C. Mullany, Alemayehu Worku, Yemane Berhane, Anne C. C. Lee

**Affiliations:** 1 Department of Pediatric Newborn Medicine, Brigham and Women’s Hospital, Harvard Medical School, 75 Francis Street, Boston, MA 02115 USA; 2 Addis Continental Institute of Public Health, Addis Ababa, Ethiopia; 3 Amhara Public Health Institute, Bahir Dar, Ethiopia; 4 Departments of Nutrition and Global Health and Population, Harvard T.H. Chan School of Public Health, Boston, MA USA; 5 Department of Obstetrics and Gynecology, Beth Israel Deaconess Medical Center, Harvard Medical School, Boston, MA USA; 6 Department of Epidemiology, Harvard T.H. Chan School of Public Health, Boston, MA USA; 7 Department of Pediatrics, Harvard Medical School, Boston, MA USA; 8 Department of International Health, Johns Hopkins Bloomberg School of Public Health, Baltimore, MD USA

**Keywords:** Low birthweight, Delivery registers, Birthweight heaping, Data quality improvement initiative, Ethiopia

## Abstract

**Background:**

Low birthweight (LBW) (< 2500 g) is a significant determinant of infant morbidity and mortality worldwide. In low-income settings, the quality of birthweight data suffers from measurement and recording errors, inconsistent data reporting systems, and missing data from non-facility births. This paper describes birthweight data quality and the prevalence of LBW before and after implementation of a birthweight quality improvement (QI) initiative in Amhara region, Ethiopia.

**Methods:**

A comparative pre-post study was performed in selected rural health facilities located in West Gojjam and South Gondar zones. At baseline, a retrospective review of delivery records from February to May 2018 was performed in 14 health centers to collect birthweight data. A birthweight QI initiative was introduced in August 2019, which included provision of high-quality digital infant weight scales (precision 5 g), routine calibration, training in birth weighing and data recording, and routine field supervision. After the QI implementation, birthweight data were prospectively collected from late August to early September 2019, and December 2019 to June 2020. Data quality, as measured by heaping (weights at exact multiples of 500 g) and rounding to the nearest 100 g, and the prevalence of LBW were calculated before and after QI implementation.

**Results:**

We retrospectively reviewed 1383 delivery records before the QI implementation and prospectively measured 1371 newborn weights after QI implementation. Heaping was most frequently observed at 3000 g and declined from 26% pre-initiative to 6.7% post-initiative. Heaping at 2500 g decreased from 5.4% pre-QI to 2.2% post-QI. The percentage of rounding to the nearest 100 g was reduced from 100% pre-initiative to 36.5% post-initiative. Before the QI initiative, the prevalence of recognized LBW was 2.2% (95% confidence interval [CI]: 1.5–3.1) and after the QI initiative increased to 11.7% (95% CI: 10.1–13.5).

**Conclusions:**

A QI intervention can improve the quality of birthweight measurements, and data measurement quality may substantially affect estimates of LBW prevalence.

**Supplementary Information:**

The online version contains supplementary material available at 10.1186/s12963-021-00265-0.

## Background

Worldwide, approximately 15% of live births (21 million) were low birthweight (LBW) in 2015; 91% from low- and middle-income countries (LMICs) [1]. LBW, commonly caused by preterm birth and/or intrauterine growth restriction [2], leads to a range of short- and long-term health impacts, including respiratory distress and feeding intolerance, growth impairment, developmental delay, and higher risk of diabetes and cardiovascular diseases [3]. LBW newborns are twenty times more likely to die in the first year than normal weight newborns [4].

Though recent estimates have shown that some progress has been made in reducing the risk of LBW [1], intensified efforts are needed to meet the World Health Assembly global target of decreasing the proportion of infants with LBW by 30% (~ 14 million) by the end of 2025. Ethiopia set a goal to reduce the prevalence of LBW to 7% by 2025 from 10.8% in 2012 [5]. Accurately measuring newborn weight at birth is crucial to provide special care for LBW infants, monitor the burden of LBW in the population, evaluate access to interventions aimed to improve antenatal care, and planning appropriate actions to accelerate the reduction of neonatal morbidity and mortality [6]. In LMICs, however, the quality of birthweight data suffers from measurement and recording errors, inconsistent data reporting systems, and missing data from non-facility births [1]. Improving measurement, recording, and reporting of birthweight are therefore warranted to target interventions and track progress toward the global nutrition target [7]. Strengthening the existing routine health systems of LMICs has been recommended as an essential strategy to improve birthweight data quality [8]. The *Every Newborn* Action Plan endorsed by the World Health Assembly prioritizes measurement improvement, with a focus on strengthening routine facility-based data, to track the national 2030 milestones (≤ 12 neonatal deaths and ≤ 12 stillbirths per 1000 live births) [9]. In LMICs, health system strengthening measures that include training of healthcare staff and supportive supervision showed to improve health facility data quality [10, 11]. The Ethiopian National Newborn and Child Survival Strategy (2015/16—2019/20) aims to strengthen the existing health information management system for improving the percentage of live births with a reported birthweight from 5.2% in 2013 to 95% by 2020 [12]. Notwithstanding, the recent global and national LBW estimates conducted by Blencowe and colleagues could not provide an estimated LBW prevalence from Ethiopia, due to lack of adequate birthweight data quality [1].

The Enhancing Nutrition and Antenatal Infection Treatment (ENAT) study is a pragmatic effectiveness study testing the impact of optimizing prenatal nutrition status and infection control on birth outcomes in rural Amhara (ISRCTN15116516). Prior to the study, we introduced a birthweight quality improvement (QI) initiative in all study sites with the objective of improving birthweight data quality. Herein, we present the impact of QI on birthweight data quality measures and the prevalence of LBW before and after the implementation of the initiative.

## Methods

### Study design and population

A comparative pre-post study was conducted to determine the effect of QI on birthweight data quality and the proportion of newborns with LBW in rural Amhara, Ethiopia. Amhara region is subdivided into 13 administrative zones and 140 districts, and it has an estimated population size of over 21,000,000. Nearly 85% of the population live in rural areas. According to the 2019 Ethiopian Mini Demographic and Health Survey (EMDHS) report, in Amhara region, the percentage of institutional delivery and those receiving antenatal care from a skilled provider were 54.2% and 82.6%, respectively [13]. Approximately 23% of women of reproductive age had a body mass index of below 18.5 kg/m^2^ [14].

### Study sites and periods

#### Phase 1 (pre-QI)

A retrospective facility record review was performed in 14 health centers located in seven districts of three zones in Amhara, namely South Gondar, West Gojjam and North Wollo, from February to May 2018 (Additional file 1). The health centers were chosen as prospective sites for the parent ENAT study, and the populations were selected based on high rates of maternal malnutrition, risk of fetal growth restriction, and need for nutrition interventions. Geomorphological maps were used to identify drought and famine-prone districts, and local government and partners were consulted. Two health centers from each district were selected based on the current antenatal care volume, using data from the region’s Health Management and Information System.

#### Phase 2 (post-QI)

After selection of the health centers for the parent ENAT study within the zones above (South Gondar, West Gojjam), birthweight QI was implemented in 12 study health centers and birthweight data were prospectively collected from late August to early September 2019, and December 2019 to June 2020. These study health facilities were selected in consultation with the Amhara Regional Health Bureau, Amhara Public Health Institute, and partners, with preference for health centers with higher ANC volume and access to transportation (Additional file 1). The phase 2 study was conducted with the objective of not only improving the quality of anthropometric measurements and recording, but also for testing study tools and procedures of the ENAT parent study.

### ***Birthweight measurement QI initiative package (Fig. ******1******)***

**Fig. 1:**
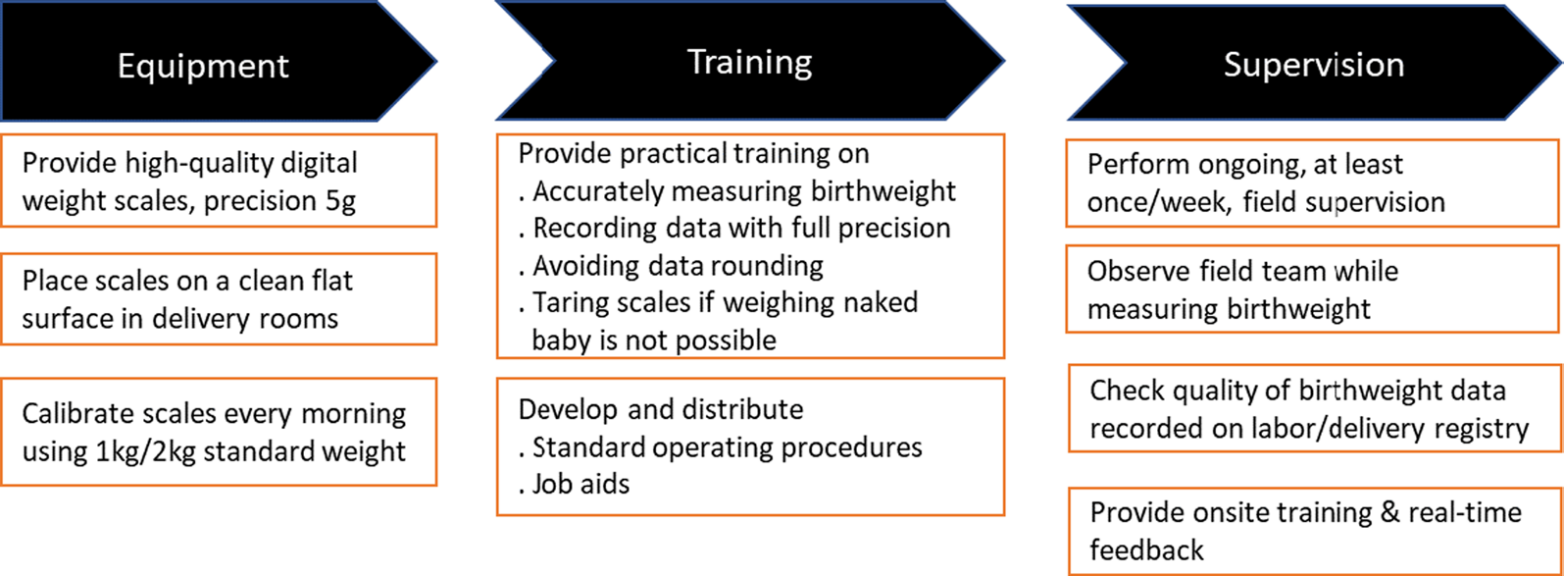
Birthweight measurement quality improvement initiative package

#### Equipment

A high-quality digital infant scale (ADE-M112600, Germany) (precision 5 g) was provided to each study health center for measuring the newborn weight at birth. Adequate weighing stations were set up within all health centers; scales were placed on a clean flat surface with the display clearly visible and calibrated every morning with the 1000 g and 2000 g standard weights. If the weight did not read between ± 10 g of the standardized weights, proper adjustments with the placement of scales were made until the correct measures were displayed.

#### Practical training

Health center staff (*n* = 1 to 4 midwives per health center) were trained on how to accurately measure birthweight using digital weight scales. Methods to weigh the baby with clothing using the tare function were developed, based on methods proposed by the International Fetal and Newborn Growth Consortium for the 21st Century (INTERGROWTH-21^st^) [15]. Where weight measurement of a naked newborn was not possible, a scale was first tared with a blanket or cloth, and weight of the baby with the clothing or blanket was taken. Training on recording the full precision of birthweight values, without any rounding to the nearest number was given to all personnel involved in measuring anthropometry. We developed job aids demonstrating how to properly measure and document birth weight using step-by-step pictorial procedures and posted them on the delivery room walls (Additional file 2).

#### Supportive supervision

Ongoing field supervision was performed in all health centers by the field team (study physicians and field coordinators) throughout the post-QI phase. To ensure the standard operating procedures were followed, the field team observed the midwives measuring and recording the newborns’ weight, checked facility birthweight records (labor/delivery registers) for heaping and rounding, and provided feedback when appropriate. In addition, a review meeting with all health center midwives and directors was held after 2 weeks of implementing the initiative (phase 2 study). Quality of birthweight data extracted from health center records, lessons learnt and ways to forward were discussed.

### Data collection and management

Birthweight data were extracted from delivery registers by trained research staff using the Survey Solutions® electronic data collection software (version 20.10, World Bank, Washington DC, USA). Routine field supervision was conducted in all sites, and collected data were reviewed and cleaned by the data management team daily.

#### Retrospective delivery register data extraction

**Fig. 2:**
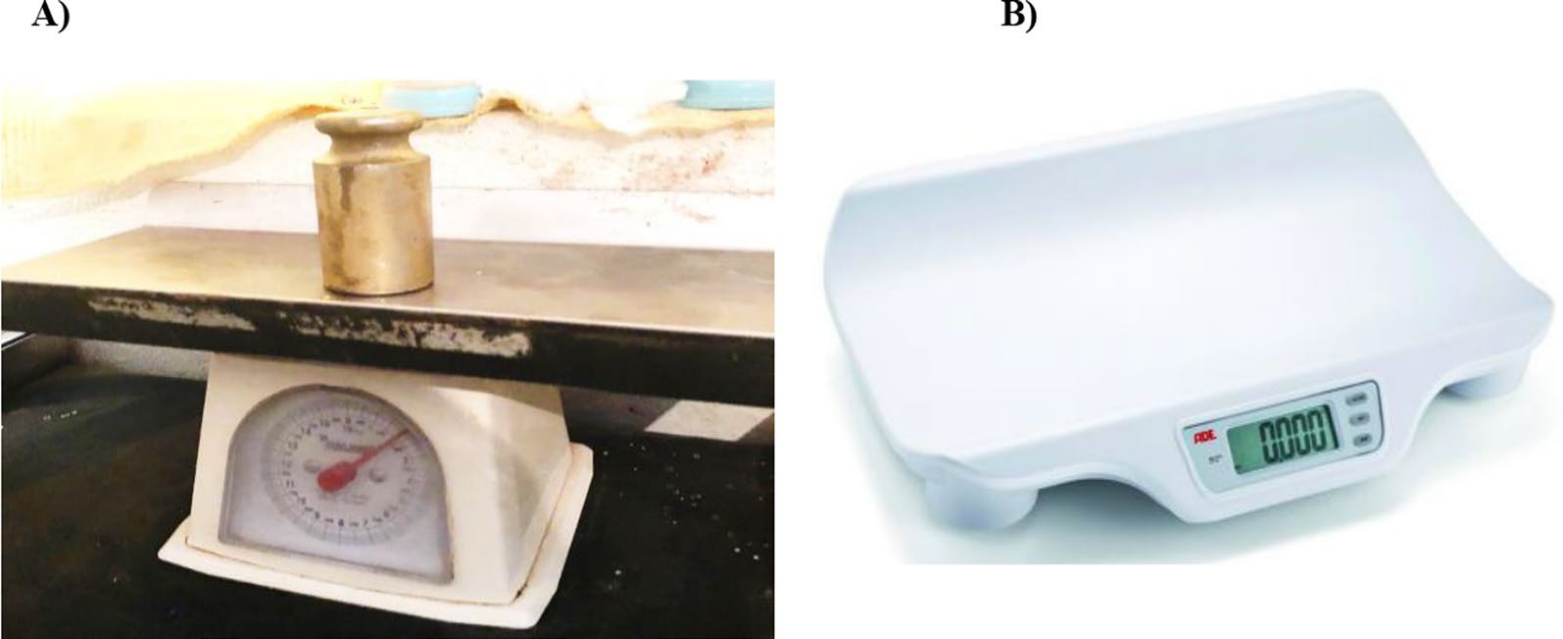
Birthweight scales used before and after initiative. **A)** Birthweight scale commonly used pre-QI. **B)** Birthweight scale used post-QI

During phase 1, about 1400 health center delivery registers were retrospectively reviewed. All labor/delivery registers recorded from February to May 2018 (*n* ~ 100 records from each health center) were included. Birthweight was measured using fully and semi-functional analog/spring weighing scales available in the health center (Fig. 2), with routine delivery room measuring practices and record keeping.

#### Prospective birthweight data collection

In phase 2, health center midwives measured the weight of newborns at birth (*n* ~ 100 per health center) using precise digital infant weight scales (Fig. 2). In line with the routine practice, health center midwives weighed the newborn only once and documented on labor/delivery registers.

### Data analyses

Birthweight data quality was assessed for implausibility, heaping and rounding. The presence of implausible values defined as extreme or unlikely birthweight values, i.e., < 350 g or > 6000 g, was checked. Percentages of heaping exactly at multiples of 500 g (e.g., 1500 g, 2000 g, 2500 g, 3000 g, 3500 g, etc.) were calculated. Heaping index (HI) was also computed by dividing the number of exact weight values (e.g., 3000 g) by all weights within the adjacent 250 g brackets, excluding the exact values (e.g., 2750–2999 + 3001–3249) [16]. Proportions of birthweights rounded to the nearest 100 g increments were calculated. Histograms were constructed to visually inspect the birthweight distribution. To calculate the prevalence of LBW, the number of live births with a birthweight of less than 2500 g was divided by the total number of liveborn babies with reported birthweights. To examine the effect of birthweight heaping, prevalence of LBW was computed by including 50% of newborns who had exactly 2500 g as LBW [16, 17]. Data analysis was done using STATA v.15 (StataCorp LLC, College Station, TX, USA).

## Results

### Quality of birthweight data

**Fig. 3:**
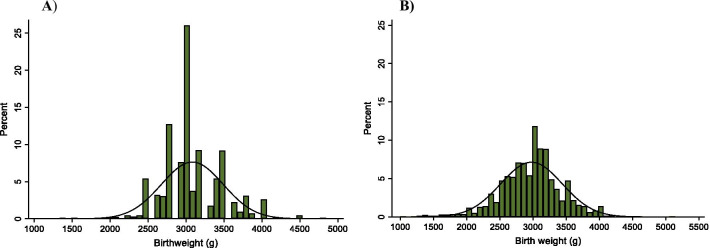
Distribution of birthweight data before and after initiative. **A)** Birthweight data pre-QI. **B)** Birthweight data post-QI

Birthweight data from 1383 and 1371 live births were collected from delivery registers in phase 1 (pre-QI) and phase 2 (post-QI) studies, respectively. There were no missing or implausible birthweights recorded at any phase. A decrease in heaping (i.e., percentages and indices of weights exactly at multiplies of 500 g) was noted across the study phases. The proportion of heaping at 3000 g was highest at 26% (HI of 0.78) before QI and dropped to 6.7% (HI 0.16) post-implementation. For the critical 2500 g at which LBW is defined, the percentage of heaping decreased by half: 5.4% pre-QI and 2.2% post-QI. About 10% of newborns were reported to have exactly 3000 g weight before the initiative, and this was reduced to 2% after the initiative. The heaping index was highest at 1.0 for 1500 g pre-QI (but only 2 observations were recorded between 1250 and 1749 g) and reduced to 0.06 post-QI (Table 1). The percentage of rounding to the nearest 100 g improved from 100% pre-initiative to 36.5% post-initiative (Fig. 3).

**Table 1:** Birthweight measurement quality before and after QI initiative in rural health centers in Ethiopia

	Pre-QI (phase 1)	Post-QI (phase 2)
Birthweights recorded	N = 1383	N = 1371
*Heaping (500 g increments)*	n (%)	n (%)
1500 g	2 (0.14%)	1 (0.07%)
2000 g	3 (0.22%)	11 (0.80%)
2500 g	75 (5.42%)	31 (2.26%)
3000 g	360 (26.00%)	92 (6.70%)
3500 g	127 (9.18%)	30 (2.19%)
*Heaping index (ratio)*		
1500 g	1.00	0.06
2000 g	0.27	0.24
2500 g	0.78	0.12
3000 g	0.78	0.16
3500 g	0.89	0.13
*Rounding*	n (%)	n (%)
Rounding to nearest 100 g	1383 (100.00%)	500 (36.50%)

### Prevalence of LBW before and after QI implementation

From health center labor/delivery registers, 2.2% (95% CI: 1.5–3.1) of newborns weighed less than 2500 g at birth pre-QI, and the percentage increased to 11.7% (95% CI: 10.1–13.5) post-QI. When half of newborns who weighed exactly 2500 g were included in the LBW calculation, the LBW rate increased to 4.9% (95% CI: 3.8–6.1) before initiative and 12.9% (95% CI: 11.1–14.7) after initiative. The proportion of newborns with 2000–2500 g also rose from 1.6% pre-QI to 9.3% post-QI (Table 2).

**Table 2:** Birthweight distribution before and after QI initiative in rural health centers in Ethiopia

Study design	Pre-QI (phase 1)	Post-QI (phase 2)	Absolute difference
	Retrospective	Prospective	
Birthweights recorded	N = 1383	N = 1371	
Birth weight	% (95% CI)	% (95% CI)	% (95% CI)
Mean (SD)	3083 (408)	2977 (463)	-106 (-138.6, -73.4)
< 2500 g	2.16 (1.5, 3.1)	11.74 (10.1, 13.5)	9.58 (7.7, 11.5)
≤ 2500 g	4.92 (3.8, 6.1)	12.91 (11.1, 14.7)	7.99 (5.9, 10.1)
2000–2500 g	1.59 (0.1, 2.2)	9.26 (7.7, 10.8)	7.67 (5.9, 9.4)
1500–2000 g	0.29 (0.01, 0.5)	1.82 (1.1, 2.3)	1.53 (0.8, 2.2)
< 1500 g	0.29 (0.01, 0.5)	0.66 (0.2, 1.1)	0.37 (-0.1, 0.9)

## Discussion

Improving quality of birthweight data is essential for identifying infants at high risk for medical and neurodevelopmental problems, determining the burden of LBW, and monitoring access and usage of interventions aimed to enhance prenatal care and newborn survival. We observed a reduction in percentages of heaping and rounding and an increase in the proportion of LBW by about 10 percentage points after the implementation of birthweight QI initiative (provision of high-precision digital weight scales, practical training and supportive supervision).

We found that introduction of QI decreased birthweight heaping exactly at multiples of 500 g (e.g., 1500 g, 2000 g, 2500 g, 3000 g, 3500 g, etc.). Heaping, commonly observed when scales with low precision are used or continuous data are rounded, represents misclassification [18]. A recent study conducted in Bangladesh, Nepal and Tanzania showed significant heaping (19–67%) at 2500 g and 3000 g in health facilities [16]. The amount of heaping on 2500 g can have a significant effect on LBW estimation. Some newborns whose birthweights rounded to exactly 2500 g may have been lighter and should actually be classified in the LBW category, while some may have been heavier. The effect of this type of misclassification is critical in determining the proportion of LBW [18]. In this study, although pre- and post-initiative data appear symmetrical, the highest percentage of heaping observed at 3000 g (26%) pre-QI declined by three-quarters after the initiative. Heaping exactly at 2500 g also reduced from 5.4 pre-QI to 2.2% post-QI. Furthermore, 100% of data rounded to the nearest 100 g pre-initiative dropped by two-thirds post-initiative. In South Africa, a similar data improvement intervention, including training on data collection and providing feedback to healthcare staff with monthly data reviews and audits, increased the accuracy and completeness of health facility data [10]. Health system strengthening measures such as performance review feedback activities and enhanced supervision have also shown to increase ownership of data among healthcare workers in Rwanda [11]. The tendency to round birthweight may be due to the fact that there are no formal standards in recording birthweight within the health systems [19], low precision and common use of spring birthweight scales [20], and/or high workload of healthcare providers. Proper recording of birthweight data on health facility records, which can be used as a source of data for regional and national estimates, may improve the quality of birthweight data and decrease the need for statistical adjustments [1].

Based on data from pre-initiative phase, the percentage of newborns with LBW was about 2%. However, the prevalence increased to 12% after birthweight QI implementation, which was more consistent with a meta-analysis that included 4105 participants from nine observational studies showing 16% of newborns were LBW in Amhara region [21]. The 2016 Ethiopian Demographic and Health Survey report indicated a LBW prevalence of 22% in Amhara region, which was the highest nationally [14]. Recent studies performed at Debre Tabor hospital in South Gondar zone, and at Dangla Primary hospital in West Gojjam zone found a LBW prevalence of 10–12% among facility births [22, 23]. Estimation of the percentage of newborns with LBW is dependent on how newborns with a reported birthweight of exactly 2500 g are classified [19]. Data from LMICs showed an increase in the prevalence of LBW from 1.7% to 7.2% after reallocating 50% of newborns with a birthweight of exactly 2500 g to the LBW category [16]. In our study, when we included half of newborns with exactly 2500 g as LBW in the calculation, the percentage increased by 2.7% pre-QI but declined to 1.2% after the initiative. In addition to recording errors, the change in estimated LBW rates due to differences in instrument precision is also possible [17]. A recent study reported lower heaping indices using digital scales compared to analog [24]. Compared to the traditional method focusing on control, audit and examination, the new approach of supportive supervision with a focus on strengthening routine health system, problem solving and training healthcare providers shows superior results in improving essential newborn care [25]. Our findings suggest that provision of digital weight scales together with periodic supervision may help to improve birthweight data quality and estimates of the true burden of LBW.

The present study has some limitations. Our findings may not be truly generalizable because women who give birth at home may differ in health and socioeconomic status from those who give birth in facilities, which may lead to different rates of LBW. Since the main aim was to improve quality of birthweight data in study health centers prior to commencing the parent ENAT study, we did not conduct proper sample size calculation and collect birthweight data from all health centers or home births throughout the study phases. Stillbirths were not routinely measured or reported and hence not included in the analysis. Given that the trial was underway, we did not repeat observations after an interval of time from the initial QI initiative, hence we could not assess whether the improvements were sustained. Despite these limitations, we were able to assess birthweight within hours of birth, prior to significant weight loss, using high-precision digital weight scales at the health facilities.

## Conclusions

Implementation of a birthweight QI program improved data quality in rural Amhara with decreased percentages of heaping and rounding on health center records. These measurement improvements resulted in an increased prevalence of LBW from 2.2 to 11.7%. Availing precise digital weight scales, improving BW recording, and birth weighing guidelines along with supportive supervision may be essential to improve birthweight data quality in low-resource settings. Further implementation research is needed to assess the effectiveness and sustainability of the initiative on a larger-scale and estimate cost implications.

## Supplementary Information

**Additional file 1**. Health centers selected for Phase 1&2 studies (pre- and post-QI).

**Additional file 2**. Job aids posted on the delivery room walls.

## Data Availability

The datasets used and/or analyzed during the current study are available from the corresponding author on reasonable request.

## Abbreviations

CI: Confidence intervals
EMDHS: Ethiopian Mini Demographic and Health Survey
ENAT Study: Enhancing Nutrition and Antenatal Infection Treatment Study
HI: Heaping Index
INTERGROWTH-21^ST^: International Fetal and Newborn Growth Consortium for the 21st Century
LBW: Low birthweight
LMICs: Low- and middle-income countries
QI: Quality improvement
