# Supplementary material for: Effect of birthweight measurement quality improvement on low birthweight prevalence in rural Ethiopia
Source: Popul Health Metr. 2021 Sep 22;19:35. doi: 10.1186/s12963-021-00265-0 (PMC8459538; doi:10.1186/s12963-021-00265-0)
Supplement: Supplementary file 1 — Additional file 1. Health centers selected for Phase 1&2 studies (pre- and post-QI). [file 12963_2021_265_MOESM1_ESM.pdf]

Additional file 1: Health centres selected for Phase 1&2 studies (pre- and post-QI) in rural Ethiopia

Table 1: Health centers selected for Phase 1 study (Pre-QI) in rural Ethiopia

Zones	Districts	Health Centers
West Gojjam	North Achefer<br>South Achefer	Blen<br>Yismala<br>Ashuda<br>Yeboden
South Gondar	Ebinat<br>Lay Gayint<br>Tach Gayint	Selamaya<br>Wergaja<br>Checheho<br>Gobegob<br>Agate<br>Aketo
North Wollo	Meket<br>Bugna	Debre Zebit<br>Serko<br>Birko<br>Kob

Table 2: Health centers selected for Phase 2 study (Post-QI) in rural Ethiopia

Zones	Districts	Health Centers
West Gojjam	North Achefer<br>South Achefer	Legdiya<br>Qunzela<br>Forhe Sankra<br>Yismala<br>Lalibela
South Gondar	Dera<br>Libokemkem	Anbessamie<br>Arb Gebaya<br>Geladiwos<br>Korata<br>Wanzaye<br>Yifag<br>Ambomeda
