# Supplementary material for: Effect of birthweight measurement quality improvement on low birthweight prevalence in rural Ethiopia
Source: Popul Health Metr. 2021 Sep 22;19:35. doi: 10.1186/s12963-021-00265-0 (PMC8459538; doi:10.1186/s12963-021-00265-0)
Supplement: Supplementary file 2 — Additional file 2. Job aids posted on the delivery room walls. [file 12963_2021_265_MOESM2_ESM.pdf]

Additional file 2: Job aids posted on the delivery room walls

INFANT WEIGHING

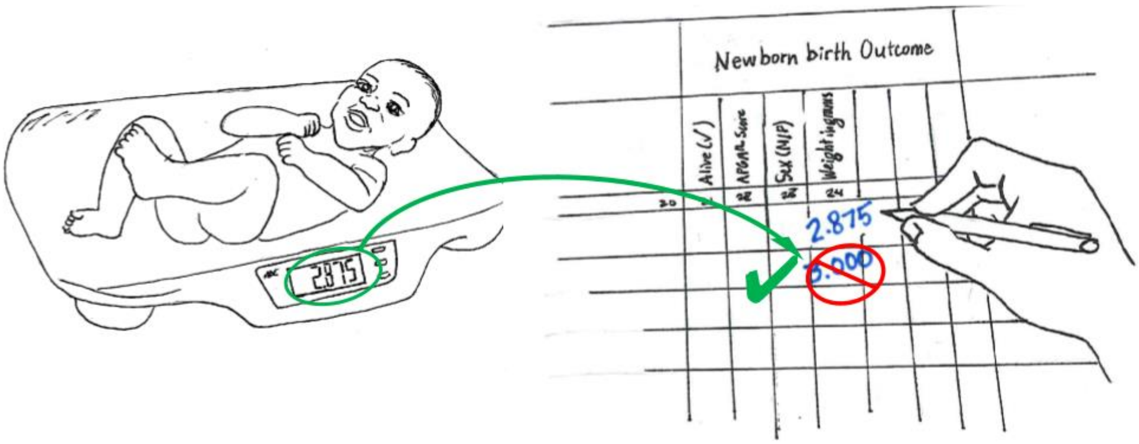

- Please precisely record all four numbers on the scale, **do NOT round up or down**
- Baby should be weighed NAKED, without any clothing/blankets on
- It is important to weigh and record weights on ALL babies born, even premature <28 or stillborn

ENAT Protocol Study Code:20190531/ENAT-0002

WEIGHING WITH A BLANKET

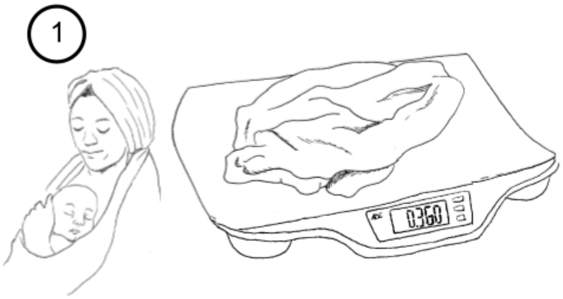

Unwrap infant and keep warm on mother's skin while you place the blanket on the scale FIRST before weighing the infant

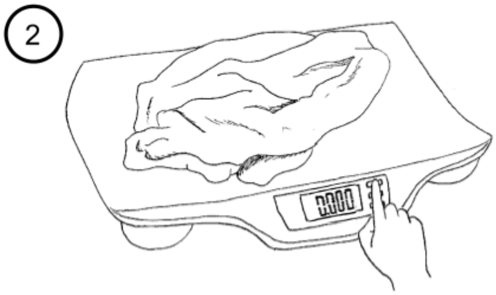

With a single finger, press the ON/TARE button to set the scale to **0.000**

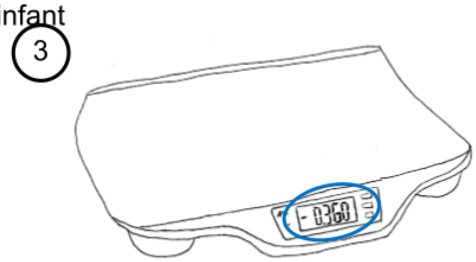

Now remove the blanket and the scale will remain NEGATIVE the weight of the blanket for several minutes

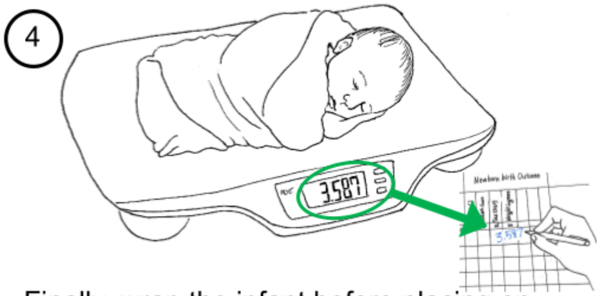

Finally, wrap the infant before placing on the scale and record the weight it reads
